# Comparative efficacy of buparvaquone and imidocarb in inhibiting the *in vitro* growth of *Babesia bovis*

**DOI:** 10.3389/fphar.2024.1407548

**Published:** 2024-05-01

**Authors:** Natalia M. Cardillo, Paul A. Lacy, Nicolas F. Villarino, J. Stone Doggett, Michael K. Riscoe, Reginaldo G. Bastos, Jacob M. Laughery, Massaro W. Ueti, Carlos E. Suarez

**Affiliations:** ^1^ Animal Disease Research Unit, United States Department of Agriculture, Agricultural Research Service, WSU, Pullman, WA, United States; ^2^ Estación Experimental INTA Paraná Consejo Nacional de Investigaciones Científicas y Técnicas (CONICET), Parana, Argentina; ^3^ Department of Veterinary Microbiology and Pathology, Washington State University, Pullman, WA, United States; ^4^ Program in Individualized Medicine, Department of Veterinary Clinical Sciences, College of Veterinary Medicine, Washington State University, Pullman, United States; ^5^ Oregon Health and Science University, Portland, OR, United States; ^6^ VA Portland Healthcare System, Portland, OR, United States

**Keywords:** *Babesia bovis*, buparvaquone, imidocarb, *in vitro* cultured parasites, efficacy

## Abstract

**Introduction:**

*B. bovis* is an apicomplexan parasite responsible for bovine babesiosis, a tick-borne disease with a worldwide impact. The disease remains inefficiently controlled, and few effective drugs, including imidocarb dipropionate (ID), are currently available in endemic areas. The objective of this study was to evaluate whether buparvaquone (BPQ), a drug currently used to treat cattle infected with the *Babesia*-related *Theileria spp.* parasites, could be active against *Babesia* parasites. Herein, we compared the effect of ID and BPQ on *B. bovis* growth *in vitro* erythrocyte culture.

**Methods:**

We compared the effect of ID and BPQ on the culture-adapted Texas T2Bo strain of *B. bovis. In vitro* cultured parasites were incubated with ID and BPQ at two starting parasitemia levels (PPE), 0.2% and 1%. *In vitro* cultured parasites were treated with ID or BPQ at concentrations ranging from 10 to 300 nM, during 4 consecutive days. Parasitemia levels were daily evaluated using microscopic examination. Data was compared using the independent Student’s t-test.

**Results and discussion:**

Both ID and BPQ significantly inhibited (*p* < 0.05) the growth of *B. bovis*, regardless of the initial parasitemia used. At 1% parasitemia, BPQ had lower calculated inhibitory concentration 50 (IC50: 50.01) values than ID (IC50: 117.3). No parasites were found in wells with 0.2% starting parasitemia, treated previously with 50 nM of BPQ or ID, after 2 days of culture without drugs. At 1% parasitemia, no parasite survival was detected at 150 nM of BPQ or 300 nM of ID, suggesting that both drugs acted as babesiacidals.

**Conclusion:**

Overall, the data suggests that BPQ is effective against *B. bovis* and shows a residual effect that seems superior to ID, which is currently the first-line drug for treating bovine babesiosis globally.

## 1 Introduction

Bovine babesiosis is a tick-borne parasitic disease with global impact on all continents (Pipano and Hadani, 1984; Bock et al., 2004), especially in tropical and subtropical areas of the world (Jacob et al., 2020; Asrar et al., 2022). Six species of Babesia are known to cause disease in cattle; however, *B. bovis*, *B. bigemina* and *B. divergens* are the species with the highest impact on the cattle industry worldwide. *B. bovis* and *B. bigemina* cause high morbidity and mortality in tropical and semi-tropical regions. In contrast, *B. divergens* has zoonotic potential and is mainly prevalent in European countries (Zintl et al., 2003, Zintl et al., 2014).

*B. bovis* and *B. bigemina* are transovarially transmitted by the one-host cattle tick *Rhipicephalus microplus* and can cause acute and persistent disease in livestock (Smith et al., 2000). Acute babesiosis is especially problematic in adult or immunocompromised cattle (Azhar et al., 2023), which also cannot be vaccinated with current live attenuated vaccines. The high direct economic effects of bovine babesiosis are associated with mortalities that may be up to 70%–90% in susceptible cattle without treatment, and secondly, with costs derived from treatments and prevention (Pipano and Hadani, 1984; Almazan et al., 2018; Jacob et al., 2020)

In *B. bovis* infections, relatively low parasitemia occur (PPE< 0.5%) in the presence of severe clinical effects in the acute stage of the disease. In addition, cattle that survive acute infection remain persistently infected for life (Alvarez et al., 2020; Azhar et al., 2023). *B. bigemina* infections, in contrast, occur with much higher parasitemias (PPE 3%) that precede severe clinical manifestations (McCosker, 1981; Bock et al., 2004; Néo et al., 2016), and infected cattle are able, in some cases, to clear the parasites after the development of a shorter persistent stage of infection. However, a critical difference between *B. bovis* and *B. bigemina* is the relative predilection of the parasitized erythrocytes of *B. bovis* to adhere to microcirculatory endothelium and thus to accumulate and sequester in blood capillaries of many organs, including the brain, causing neurological syndrome (Kakoma and Ristic, 1984; Böse et al., 1995). Considering this parasite sequestration phenomenon, the disease usually progresses very fast to circulatory collapse, respiratory failure, coma, and death (Kakoma and Ristic, 1984), and thus *B. bovis* is considered the most pathogenic agent of bovine babesiosis.

Bovine babesiosis can be currently controlled by strategies targeting the tick vectors, the use of live vaccines, and babesiacidal drugs, such as diminazene aceturate, amicarbalide, and imidocarb (Todorovic et al., 1973; Vial and Gorenflot, 2006; Almazan et al., 2018; Alvarez et al., 2020; Azhar et al., 2023). However, current live vaccines are not fully safe and require a cold chain, among other limitations. The drugs against ticks for treatment are expensive and may lead to acaricide resistance, have toxic effects, and leave residues in the food chain. Thus, new approaches for controlling the disease are urgently needed (Pérez de León et al., 2010; Pérez de Leon et al., 2012; Almazan et al., 2018).

In addition, vaccination of less than year-old calves using live attenuated parasites, one of the main strategies for babesiosis control and prevention, needs to be integrated strategically to maintain enzootic stability in endemic areas (Smith et al., 2000; Asrar et al., 2022). Chemotherapy against bovine babesiosis is an important component in the control of the disease and several drugs and drug combinations have been reported to be effective against the parasite (Kuttler and Aliu, 1984; Silva et al., 2018; Silva et al., 2020; Li et al., 2021; Rizk et al., 2023). Treatment success is dependent on early diagnosis, the severity of the disease, the dosage used, the timing of treatment in the course of infection, and the length of time that the drug is present to affect the parasite (Mehlhorn, 2016). Large morphological *Babesia* species, such as *B. bigemina*, were shown to be more susceptible to antiprotozoal drugs than smaller species (such as *B. bovis*), but no treatment by itself can be relied upon to give a radical cure (Vial and Gorenflot, 2006; Mehlhorn, 2016).

Imidocarb dipropionate (ID) is considered the first-line treatment for bovine babesiosis and remains the most used chemotherapeutic measure worldwide. However, ID is only approved for *Babesia* treatment in horses (NADA 97-288) and dogs (NADA 141-071), but not for cattle in the US (CVM- FDA, 1997). This drug has a potent babesiacidal effect that consistently clears the parasites from the host (Lewis et al., 1981), and has been used as a prophylactic drug protecting against clinical diseases from 3 to 6 weeks. As the drug level decreases, it allows mild subclinical infection to occur, resulting in premunition and immunity (Kuttler, 1981; Kuttler et al., 1975; Todorovic et al., 1973), a feature that is especially important in endemic areas (Vial and Gorenflot, 2006). Clinical relapses or decreases in the efficacy of ID have been reported (Sears et al., 2020). The mechanism of action of ID is not clearly understood (Todorovic et al., 1973), but two possible modes of action have been proposed, including blocking inositol entry into *Babesia*-infected erythrocytes resulting in parasite starvation (McHardy et al., 1986), or binding to nuclear DNA in susceptible *Babesia* species causing nucleic acid damage, inhibiting cellular repair, and replication of the parasites (Baneth, 2018; Rizk et al., 2023).

In addition, ID may have toxic secondary effects in treated animals at therapeutic doses (Todorovic et al., 1973; Adams et al., 1980; Vial and Gorenflot, 2006) and is retained in edible tissues of cattle for prolonged periods after treatment (more than 7 months)(Coldham et al., 1994; Tang et al., 2023). These limitations suggest that, although effective as babesiacidal, ID is not an ideal drug for use in a *Babesia* control program due to its potential toxicity in animals and its persistence in the foodstuff derived from treated animals, and alternative new medicines are needed.

BPQ is a second-generation hydroxynaphthoquinone antiprotozoal drug originally developed as an anti-malarial compound (Hudson et al., 1985)that has also been studied *in vivo* and *in vitro* for the treatment of *Theileria* species in bovines(Wilkie et al., 1998; Muraguri et al., 2006; Carter, 2011; Bailey, 2013; Goud et al., 2021). BPQ is currently considered the drug of choice in endemic regions against theileriosis in cattle (Ibrahim et al., 2020). However, despite the similarities between *Theileria* and *Babesia* (both members of the order piroplasmida), we found only one previous report about the *in vitro* effect of BPQ on *Babesia* spp. parasites.

BPQ acts selectively against the parasite’s electron transport system at ubiquinone-cytochrome bc_1_ complex, leading to competitively inhibiting the parasite’s electron transport respiratory chain but not in the vertebrate host at the parasite inhibitory levels. This mechanism of action also prevents the parasite from taking up pre-formed pyrimidines to relieve the blockade, resulting in a lethal effect (Hudson et al., 1985; Fray and Pudney, 1992; Sharifiyazdi et al., 2012; Mhadhbi et al., 2015).

BPQ is registered in about 20 countries around the world for the treatment of East Coast fever and tropical theileriosis (Carter, 2011), but it is not registered in many others, such as the United States, European Union countries, and Australia, due to the persistence of chemical residues that could impact export markets (Müller et al., 2016), so maximum residue levels (MRL) for the drug have not been established. However, new analytical methods demonstrated in dairy cattle treated at a single time with 2.5 mg/kg of BPQ, that the drug could be detected (LOD; 0.005 mg/kg) in milk for at least 35 days post-treatment and in the liver and injection site to at least 328 days post-treatment (McDougall et al., 2016).Similar to ID, the efficacy of BPQ as an antiparasitic drug could be related to the persistence of the drug in cattle tissues for long periods of time.

Based on these preliminary observations and the fact that *Theileria* and *Babesia* are related intra-erythrocytic apicomplexan parasites of cattle, the aim of this study was to study the efficacy of BPQ on its ability to inhibit the *in vitro* growth of *B. bovis,* in comparison with ID.

Overall, the data emerging from this study indicates that BPQ is effective in controlling the growth of *B*. *bovis* in *in vitro* cultures, even at lower drug concentrations than ID, and supports further *in vivo* studies aimed at confirming the potential for the application of BPQ in babesiosis control programs.

## 2 Materials and methods

### 2.1 Chemical reagents

BPQ (98% pure) was obtained from Combi-Blocks, Inc. (San Diego, CA, United States). ID (VETRANAL™, Supelco^®^ Buchs, Switzerland) was used as a positive control for the *in vitro* inhibition assays for *B. bovis*, using an identical protocol as BPQ described below. Purity of ID was determined to be >98% by proton-nuclear magnetic resonance spectroscopy and High-performance liquid chromatography (HPLC), according to the certificate of analysis.

BPQ and ID were diluted in 100% dimethyl sulfoxide (DMSO) to prepare stock solutions, which were kept at room temperature until use. Working solutions were freshly prepared in a parasite culture medium every test day before being added to the parasite cultures.

### 2.2 Parasites culture

*B. bovis* (Texas T2Bo strain: Goff et al., 1998) were grown in long-term microaerophilic stationary-phase cultures and incubated at 37°C in an atmospheric condition of 5% CO_2_, 5% O_2_, and 90% N_2_, as previously described (Levy and Ristic, 1980). *B. bovis* were cultured in 96-well-culture plates, in 180 μL per well of PL culture media (100 mL = pH 7.2; 29 mL F-12K Nutrient Mixture + L-glutamine Life Technologies, 29 mL Stable Cell IMDM Sigma Aldrich, 2 mL 0.5 M TAPSO Sigma Aldrich, 0.5 mL Antibiotic Antimycotic solution 100X Sigma Aldrich, 1 mM calcium chloride Sigma Aldrich, 100 μL Antioxidant Supplement 1000x Sigma Aldrich, 1 mL Insulin-Transferrin-Selenium-Ethanolamine 100x Sigma Aldrich, 1 mL 50% Glucose Teknova, 500 μL L-glutamine 200 mM GIBCO), supplemented with 40% bovine serum and contained a suspension of 10% cells volume of erythrocytes (RBCs).

### 2.3 *In vitro* growth of initial inhibitory assay

The *in vitro* inhibitory efficacies of BPQ and ID were evaluated against the growth of the T2Bo *B. bovis* strain, with a starting percentage of parasitized erythrocytes (PPE) of 0.2% and 1%. *B. bovis* were grown, as described above, in culture media containing different concentrations of ID and BPQ: 10, 25, 50, 100, 150, 200, and 300 nM, diluted in 100% DMSO. Parasites culture in the presence of DMSO (1 μL) and the absence of drug compounds were used as a positive control for parasite growth. Extra wells containing uninfected bovine RBC were prepared and used as negative controls. Parasite cultures were fed daily with fresh culture medium (180 μL/well) containing the respective drug concentration. The experiments were carried out in triplicate for each tested concentration and control, over 96 h (4 days). PPE was monitored daily by counting parasites in Hema 3 Stat Pack (Fisher Scientific) stained thin blood smears (GBS). Before the daily change of the media, the supernatant media (180 μL) of each well was extracted, and the bottom with the RBC layer was homogenized; 1 μL of sample was taken from each well to make a thin smear, and the number of infected red blood cells was counted by visual examination of 5000 erythrocytes in each slide. Morphological appearance was also recorded. Drug responsiveness of the parasites was measured as percentage parasitemia after every 24 h exposure to each concentration of drug.

### 2.4 Viability after treatment

At 96 h after the last treatment, fresh medium without drug was replaced in all the culture wells and 10 μL of fresh RBCs were added. The same procedure was done for the next 2 days to determine if the culture was viable and could continue growing in the absence of the drug. Quantitative and qualitative parasitemia was determined by microscopic examination of Hema 3 Stat Pack (Fisher Scientific) stained thin blood smears.

### 2.5 Statistical analysis

Values of parasitemia were compared using independent Student’s t-test. The dose of a drug that produces 50% inhibition (IC50) relative to the control population, and the maximal (100%) inhibitory concentration (IC100) were estimated for BPQ and ID at concentrations ranging from 10 to 300 nM, at 24 h, 48 h, 72 h, and 96 h of incubation. Total inhibitory concentrations (IC100) were calculated as the doses of drug required to inhibit parasite growth to an identical level as found for non-infected erythrocytes (approximately 0.1%). The survival curves PPE 0.2% and 1% treated with imidocarb or buparvaquone were compared using Log-rank (Mantel-Cox) test. The level of significance was set at <0.05. GraphPad Prism ver. 7 software for Windows (Graphpad Software Inc., San Diego, CA, United States) was used for the statistical analysis.

## 3 Results

### 3.1 Comparative *in vitro* growth inhibitory effects of *B. bovis* by BPQ and ID

**FIGURE 1:**
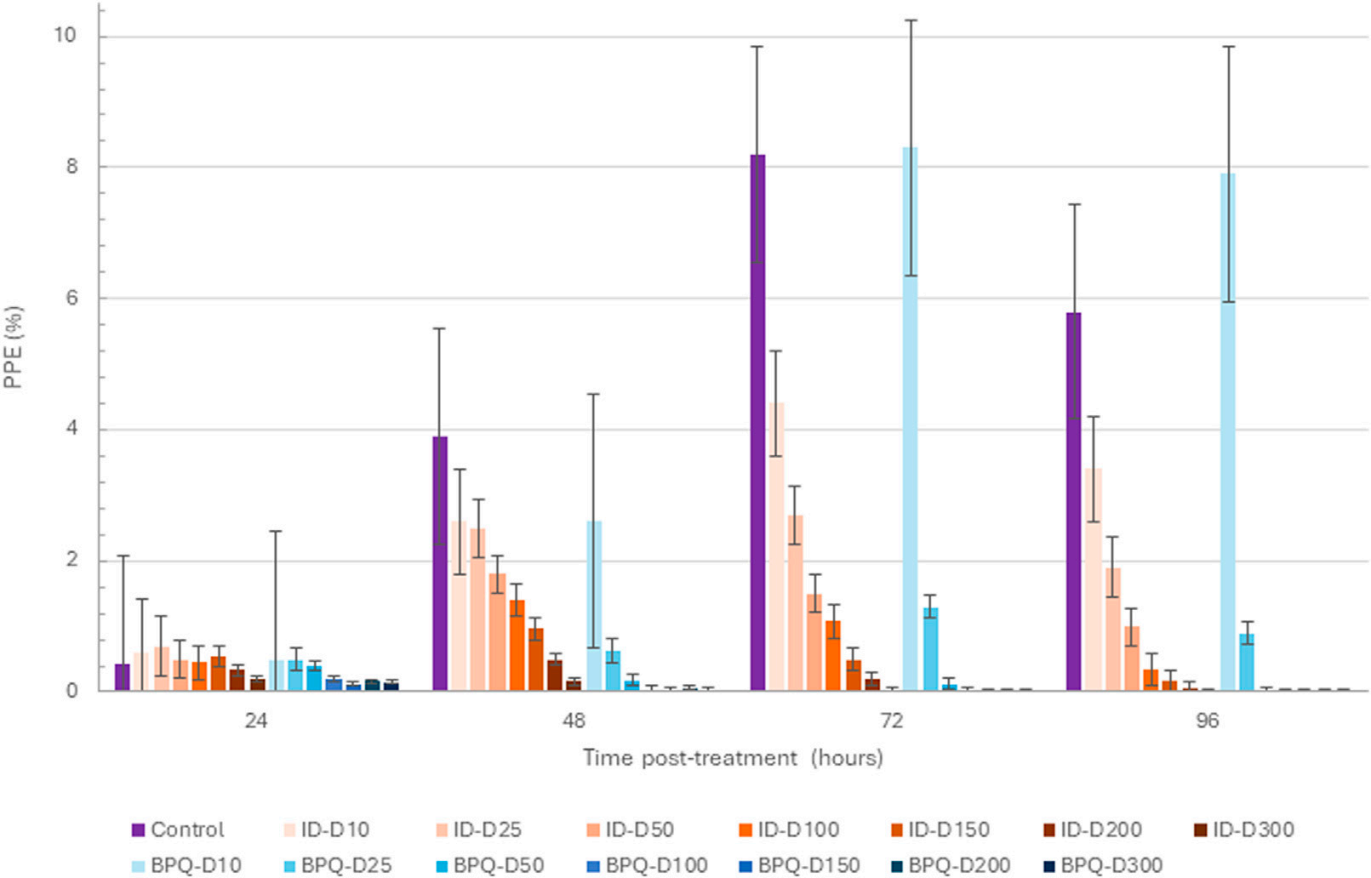
*B. bovis* T2Bo strain *in vitro* culture growth (starting PPE 0.2%) at 24, 48, 72, and 96 h in the control group, and after the addition of different concentrations of ID (orange gradients bars) and BPQ (blue gradient bars). Error bars indicate the standard error of the means for each BPQ and ID concentration (nM) tested.

Parasite growth *in vitro* culture up to 96 h of incubation, starting with 0.2% PPE to BPQ and ID, are depicted in Figure 1 and Table 1. While BPQ appears to be non, or poorly effective, at 10 nM, ID is significantly more effective at this low concentration. In contrast, BPQ appears to be more effective than ID at all the other concentrations tested after 48 h in culture (Figure 1). Among all treatments applied, the differences in the median percentages of survival between BPQ and ID were statistically different (Table 1). However, while no parasite survival was found at 50 nM and above for BPQ, this is only the case for ID at 300 nM, suggesting that BPQ has higher efficacy than ID for eliminating the parasite under the experimental conditions tested.

**TABLE 1:** Comparative survival (%) of *in vitro B. bovis* Texas strain culture at 0.2% PPE in the presence of different concentrations of buparvaquone and imidocarb at 96 h post-treatment.

Concentration (nM)	ID		BPQ		*p*
	Mean (%)	95% CI	Mean (%)	95% CI	
10	55.37	(44.34–66.40)	129.47	(108.60–150.35)	***p < 0.01***
25	33.56	(22.53–44.58)	19.02	(2.17–35.86)	***p < 0.05***
50	17.34	(14.93–19.74)	0	0	***p < 0.01***
100	6.38	(1.96–10.79)	0	0	***p < 0.01***
150	3.08	(1.02–5.13)	0	0	***p < 0.01***
200	1.09	(-0.48 - 2.66)	0	0	***p < 0.05***
300	0	0	0	0	n/a

**FIGURE 2:**
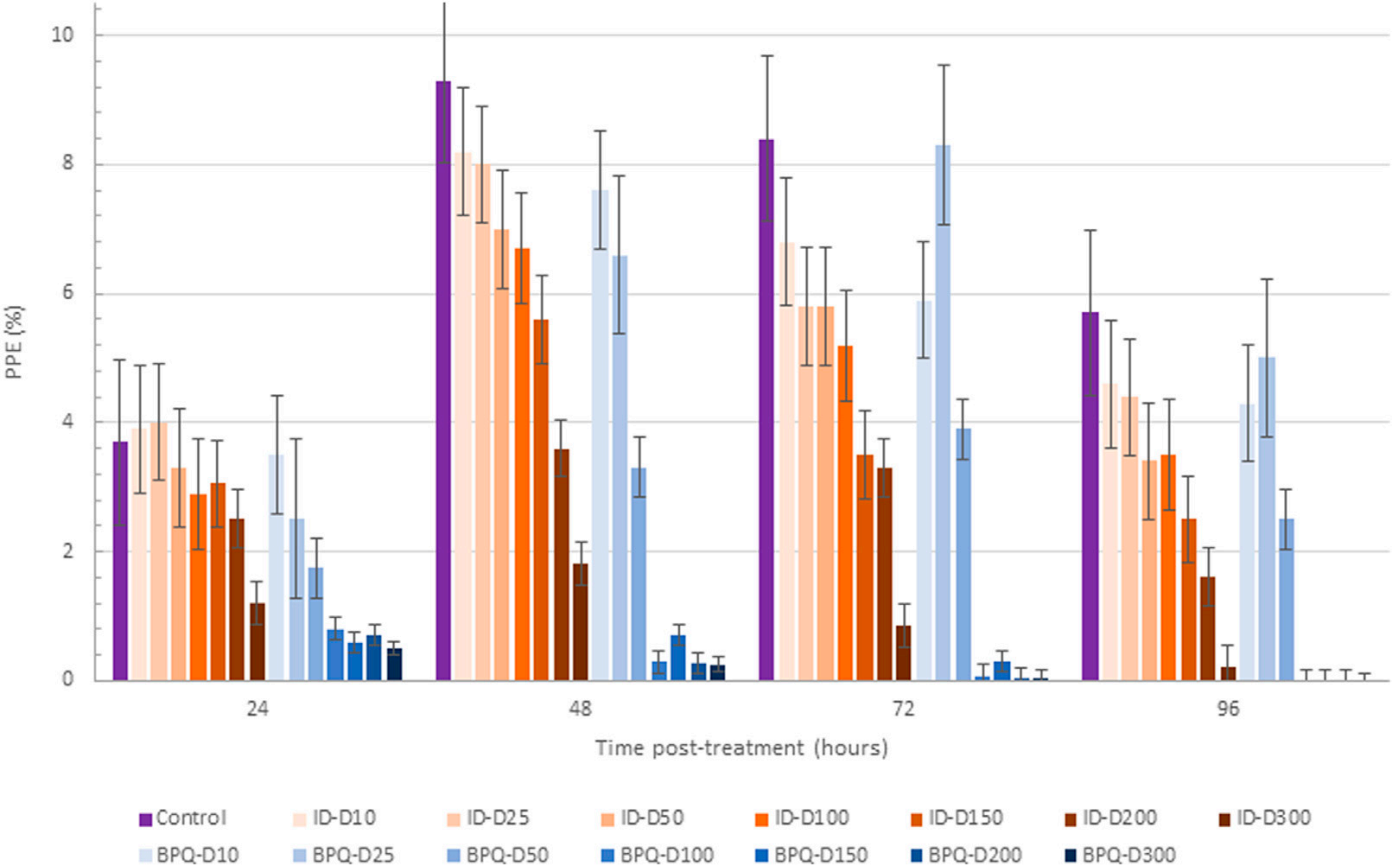
*Babesia bovis* T2Bo strain *in vitro* culture growth (starting PPE 1%) at 24, 48, 72, and 96 h in the control group, and after the addition of different concentrations (nM) of ID (ID: blue gradients bars) and BPQ (BPQ: orange gradient bars).

The effect of ID and BPQ on parasite growth in cultures starting at 1% PPE are shown in Figure 2. The growth rate in ID-treated cultures gradually and consistently decreased over time in a dose-dependent manner. In contrast, parasites treated with 10 nM and 25 nM BPQ seem to grow at rates similar to those of the control non-treated parasites. However, the growth rate of the parasite decreases sharply at concentrations higher than 50 nM of BPQ. The parasitemia data presented in Table 2 consistently showed no significant differences in survival rate for parasites exposed to 10 or 25 nM BPQ, but significant differences at BPQ concentrations higher than 25 nM. Importantly, under these experimental conditions, no parasites were found at BPQ concentrations of 150 nM and above (Table 2). In contrast, full elimination of the parasite with ID was not achieved at any of the conditions tested herein.

**TABLE 2:** Comparative survival of *in vitro B. bovis* Texas strain culture at 1% PPE in the presence of different concentrations of BPQ and ID at day four post-treatment.

Concentration (nM)	ID		BPQ		*p*
	Mean	95% CI	Mean	95% CI	
10	86.16	(46.30–126.03)	82.55	(70.22–94.88)	*p* = 0.7
25	83.65	(76.49–90.81)	98.19	(75.20–121.18)	*p* = 0.06
50	69.81	(41.30–98.32)	50.03	(36.58–63.49)	*p* = 0.05
100	64.15	(56.03–72.27)	0.5	(-1.65 - 2.65)	***p < 0.01***
150	47.80	(40.64–54.96)	0	0	***p < 0.01***
200	31.45	(26.03–36.86)	0	0	***p < 0.01***
300	3.4	(1.77–5.02)	0	0	***p < 0.01***

Overall, in all cases, the final PPE across the range of drug concentrations tested reflected a dose and time-dependent effect for both drugs tested in these experiments. Also, BPQ appears to be more efficient in rendering non-viable parasites regardless of the initial PPE used in the cultures.

### 3.2 Drugs potency

The mean and range IC50 were calculated to compare drug potency on the growth of *in vitro B. bovis* T2Bo strain cultures. At 1% PPE, means and ranges IC50 were 50.01 (48.75–50.81) and 117.3 (77.35–140.2) for BPQ and ID, respectively. At 1%, BPQ showed statistically higher potency than ID (*p* < 0.05). At 0.2% PPE, it was not possible to fit a model to estimate the IC50 for BPQ, given the extraordinary lethality of BPQ under these experimental conditions; meanwhile, for ID, the mean and range IC50 was 12.99 nM (12.50–13.46), respectively.

### 3.3 Time of survival under treatment

At 0.2% PPE, BPQ was 100% lethal on the 4th day (96 h) in cultures treated with 50 nM, and at 3.5 days in culture media treated with concentrations between 100 and 300 nM (Table 1). These results were statistically different (*p* < 0.05) compared to ID (*p* < 0.01), which only demonstrated 100% lethality at the higher concentration of 300 nM at 96 h post-treatment (Table 1). Similar results were observed at 1% PPE, where BPQ was 100% lethal at 4 days in culture media treated with concentrations between 100 and 300 nM, and this was statistically different than media treated with ID (*p* < 0.01), in which 100% lethality could not be demonstrated, even with concentration doses of 300 nM (Table 2).

### 3.4 Parasite recrudescence after treatment

Considering 0.2% starting parasitemia treated previously with doses of 50 nM of BPQ and ID, parasites were no longer viable or capable of growth after 2 days of culture without drugs (Table 1). At 1% starting parasitemia, no parasite relapse was found in treatments at doses of 150 nM of BPQ and 300 nM of ID (Table 2).

**FIGURE 3:**
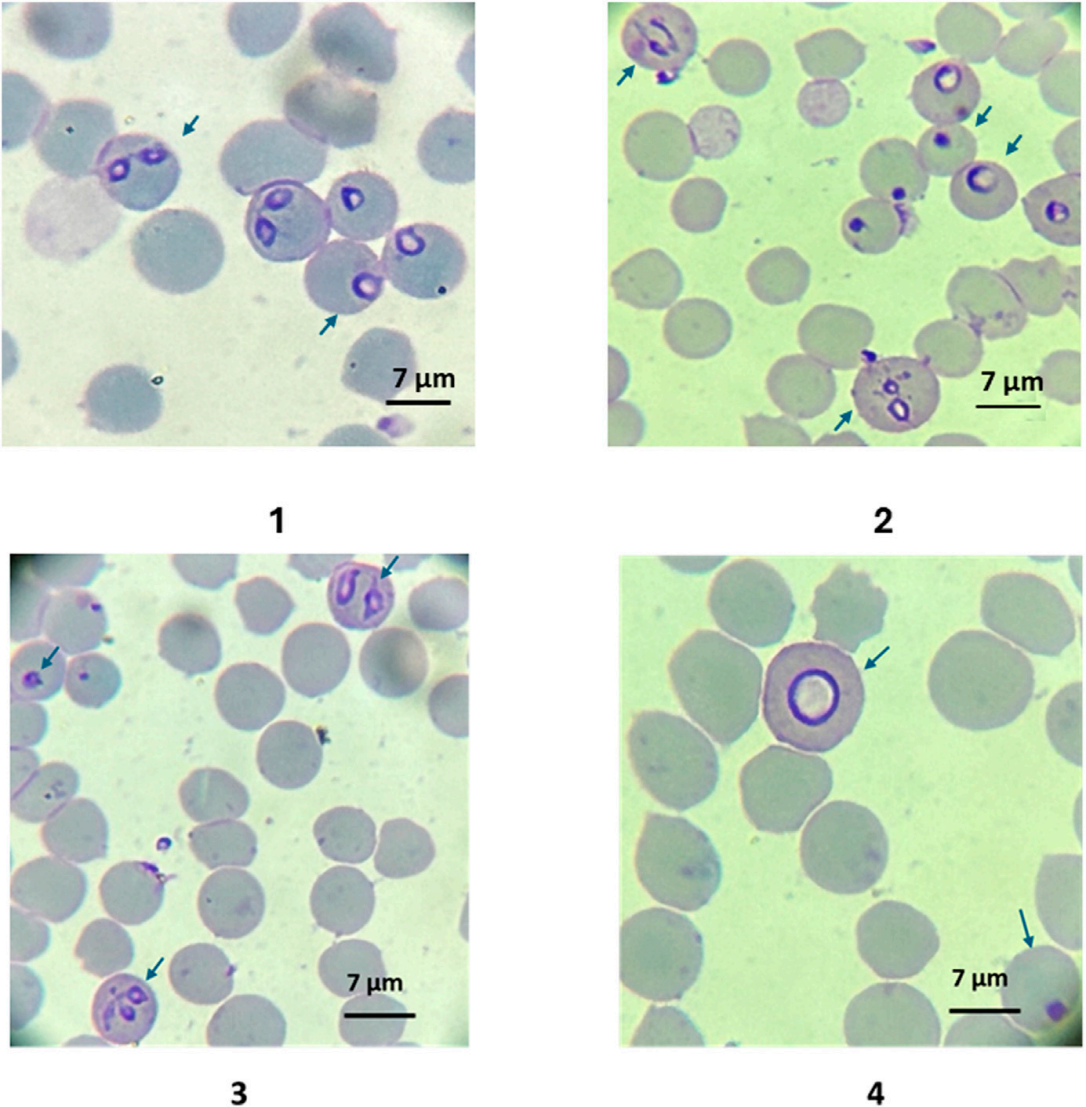
1: Typical *B. bovis* blood stage morphologies (pear shaped merozoites and rounded trophozoites) of *in vitro* cultured parasites in control group at day 3; 2: Morphologies of *B. bovis* blood stage parasites (amorphous, dotted, and enlarged round forms) in cultures treated with ID 200 nM at day 3. Morphologic characteristics of *in vitro* cultured *B. bovis* treated with BPQ 200 nM on day 2 of culture. Dot-shaped parasites and amorphous pear-shaped stages within the red blood cells. 4. Detailed image of a large round shaped intraerythrocytic parasite. Slides stained with Hema 3 stain (100X). The arrows show the infected red blood cells.

Parasites treated with 10–300 nM have noticeable morphological differences compared to the non-treated control. Single trophozoite stages were bigger and rounded in shape, and paired pear-shaped stages appeared enlarged or amorphous. The remnants of parasites within the red blood cells appeared as small pycnotic dots (Figure 3).

## 4 Discussion

A main challenge for the chemotherapeutic control of bovine babesiosis is the limited number of drugs that are effective against *Babesia* parasites, with most of them not registered in many countries or approved for specific implementation by the national regulatory agencies (Azhar et al., 2023). In addition, several reports show variable efficacy to ID or clinical relapses against certain species of *Babesia* (Pudney and Gray, 1997; Kumar et al., 2003; Checa et al., 2017). This poses a serious problem, considering that ID is considered as the drug-of-choice against bovine babesiosis. Therefore, further studies should be conducted to identify new compounds with effective babesiacidal properties. We pose hereby that buparvaquone (BPQ), a theilericidal drug, is a viable Babesiacidal that can be an alternative to ID. Importantly, BPQ was previously shown to be effective against B. bovis and B. bigemina in in vitro testing and nontoxic for vertebrates (McHardy, 1990; McHardy et al., 2010; Müller and Hemphill, 2016; Checa et al., 2017; Isik et al., 2018; Rufener et al., 2018) at the treatment doses used, environmentally safe, and already approved for treatment of bovine theileriosis in several countries.

In this study, we compared the efficacy of ID and BPQ using *in vitro* B. bovis culture. These are the second *in vitro* results reported on the effects of BPQ against B. bovis (Nugraha et al., 2019). ID is relatively effective to treat cattle babesiosis, but it is far from an ideal drug due to the aforementioned severe side effects in cattle (Todorovic et al., 1973; Adams et al., 1980; Vial and Gorenflot, 2006). In addition, ID leaves undesirable long-term residues in food (Coldham et al., 1994; Tang et al., 2023). Even more, although ID drug resistance has not been officially reported yet in B. bovis, it is likely that resistance will develop over time. On the other hand, BPQ has been approved for use in cattle against Theileria parasites in several countries and has a better safety and tolerance record compared to ID in cattle and other animal species (McHardy, 1990; Kumar et al., 2003; Müller et al., 2016; Checa et al., 2017; Isik et al., 2018; Tahir et al., 2020), making this drug, if effective, a viable therapeutic option for treating B. bovis infections.

The efficacy *in vitro* of ID and BPQ against B. bovis was also addressed in previous studies. The DL50 of ID on B. bovis T2Bo was found hereby to be between 45,55 nM–50 nM (around 1.59 × 10^−5^ mg/mL to 1.74 × 10^−5^ mg/mL), at 48–96 h post-treatment respectively. These values were lower than those reported by Brasseur et al. (1998) on B. divergens (human and bovine isolates *in vitro* cultures), who reported a DL50 of 77 (2.7 × 10^−5^ mg/mL) and 97 nM (3.4 × 10-5 mg/mL), respectively (Brasseur et al., 1998), using a tritiated hypoxanthine incorporation assay to evaluate the PPE at 48 h post-treatment. Although the IC50 values obtained with the tritiated hypoxanthine incorporation assay were higher than those obtained by microscopic examination, these differences were found not to be statistically significative.

Also, the effect of Imidocarb was also tested in other Babesia-related species. A study performed by Hines, in Theileria equi, with a starting PPE of approximately 0.5% and using flow cytometry to evaluate parasite infection of erythrocytes, reported and IC50 of 24 nM, which is higher than the IC50 values for ID found for B. bovis in the current study (13 nM). Interestingly, in the same study, complete growth inhibition was achieved when the PPE remained at the starting level of 0.3%–0.5% after 72 h, indicating no active parasite growth. In addition, Hines et al. did not determine the IC90 at 72 h post-treatment because, even at the maximum drug concentration (775 nM), the PPE still reached 1.2%, reflecting a lack of complete growth inhibition for this T. equi strain (Hines et al., 2015). In our B. bovis study, the parasites were no longer viable or capable to growth after 2 days of culture without ID upon treatment at 300 nM for 96 h (4 days)

On the other hand, we demonstrated hereby that B. bovis T2B strain parasites were not able to growth after 96 h of culture, with the addition of 100 nM, 150 nM, 200 nM, and 300 nM of BPQ, regardless of their initial PPE. However, at 0.2% initial parasitemia, 100% lethality was seen with BPQ at lower doses (50 nM). Importantly, no surviving parasites were detected after 2 days of culture without the drug in cultures derived from the wells treated previously with BPQ from concentrations 50 nM at 0.2% PPE, and from 150 nM at 1% PPE. Interestingly, a previous report on the effect of BPQ on B. bovis (Nugraha et al., 2019), reported IC50s for BPQ of 135+/−41 nM after 96 h of B. bovis culture at 1% starting PEE and 488+/−30 nM for B. bigemina; their reported IC50 for B. bovis was twice the IC50 obtained in our study (IC50: 50.01 (48.75–50.81) (Nugraha et al., 2019). However, the Nugraha et al. study uses distinct experimental conditions (including the method for parasite quantification), and B. bovis strain, precluding thorough comparisons with the current study.

Overall, the findings in this study suggest that BPQ represents a promising candidate as a new babesiacidal agent. This is supported by several lines of evidence in our study: 1] At 0.2% and 1% PPE, BPQ was 100% cidal at 4 days (96 hs) in cultures treated with concentrations between 100 and 300 nM; 2] No parasite growth was found after 2 days of incubation without replacing the drug during daily media changes at doses 150 nM; 3] The performance of BPQ was superior to ID, which could not attain 100% lethality even at 300 nM after 4 days of treatment; and 4] At higher PPE (1%), BPQ potency (IC50) 50.01 (ranging from 48.75 to 50.81) was about twice the potency of ID 117.3 (ranging between 77.35–140.2). Furthermore, regardless of the potency comparison of drugs, the calculated IC50 for BPQ against *B. bovis* was two times lower than ID, and much lower than the values previously reported for ID (Rodriguez and Trees, 1996) and also BPQ (Nugraha et al., 2019). A residual effect against parasites was seen after treatment with BPQ and ID. Post-antibiotic effect (PAE) is described for some antimicrobial agents, as the suppression of bacterial growth following a limited period of exposure to an antibiotic and removal of the compound (Proma et al., 2020), and it has been proposed as an explanation for the success of intermittent dosing regimens. Depending on the drug concentration and susceptibility of the target organisms, PAE may last for several hours post-administration (Zhanel et al., 1991). Applying this concept to apicomplexan parasites, these observations suggest that both BPQ and ID can have a persistent effect comparable to the PAE described for some antibiotics. The exact reason explaining this finding remains to be determined. It is possible to speculate that the extent of the damage or metabolic change precludes parasite recovery after 4 days of exposure to the drug. The clinical impact of this observation also deserves further investigation. In this study, we found that the inhibitory effect of both drugs was time and dose-dependent since the PPE decreased upon longer exposures, and no growth of parasites was observed with increased concentrations of drugs. Dose and time-dependence drug results agree with previous studies on *B. bovis* testing endochin-like quinolones, tulathromycin drugs (Silva et al., 2018; Silva et al., 2020), and artemisinin (Mazuz et al., 2013). In contrast, the results were independent of the starting PPE in both previous studies. A dose-dependent effect was observed in *Theileria* exposed to ID (Hines et al., 2015).

However, although studying the potency of drugs using an *in vitro* approach may be a rationale predictor of their therapeutic potential *in vivo*, the approach has several limitations, including differences among laboratories and parasite strains (Alzan et al., 2022). Many other host-related factors, such as immune response, drug metabolism and pharmacokinetic behavior, do not play a role in *in vitro* settings (Sim and Kauser, 2016), making *in vivo* testing a requirement for drug validation.

In summary, the results presented here, through the *in vitro B. bovis* culture evaluation of Buparvaquone and Imidocab, demonstrate an overall higher efficacy of BPQ at inhibiting *B. bovis,* than ID. Taking together previous *in vivo* safety and efficacy studies performed in other apicomplexan parasites that can infect animal species and humans, and the data generated hereby, BPQ emerges as an optimal candidate for *in vivo* evaluation as an alternative drug for the treatment of bovine babesiosis caused by *B. bovis*. Future work will address the potential of BPQ as a babesiacidal drug in *in vivo* studies.

## 5 Conclusion

The data collected in an *in vitro* testing model using cultured parasites suggests that BPQ is effective against *B. bovis,* displaying a residual effect that seems superior to ID, which is currently the first-line drug for treating bovine babesiosis globally. However, the potential of BPQ as a babesiacidal drug remains to be assessed using an in *in vivo* infected bovine model.

## Data Availability

The raw data supporting the conclusion of this article will be made available by the authors, without undue reservation.
